# The age and evolution of an antiviral resistance mutation in *Drosophila melanogaster*

**DOI:** 10.1098/rspb.2007.0611

**Published:** 2007-06-05

**Authors:** Jenny Bangham, Darren J Obbard, Kang-Wook Kim, Penelope R Haddrill, Francis M Jiggins

**Affiliations:** School of Biological Sciences, Institute of Evolutionary Biology, The University of Edinburgh, Ashworth LaboratoriesThe King's Buildings, West Mains Road, Edinburgh EH9 3JT, UK

**Keywords:** sigma virus, *Drosophila melanogaster*, *ref(2)P*, host–parasite coevolution

## Abstract

What selective processes underlie the evolution of parasites and their hosts? Arms-race models propose that new host-resistance mutations or parasite counter-adaptations arise and sweep to fixation. Frequency-dependent models propose that selection favours pathogens adapted to the most common host genotypes, conferring an advantage to rare host genotypes. Distinguishing between these models is empirically difficult. The maintenance of disease-resistance polymorphisms has been studied in detail in plants, but less so in animals, and rarely in natural populations. We have made a detailed study of genetic variation in host resistance in a natural animal population, *Drosophila melanogaster*, and its natural pathogen, the sigma virus. We confirm previous findings that a single (albeit complex) mutation in the gene *ref(2)P* confers resistance against sigma and show that this mutation has increased in frequency under positive selection. Previous studies suggested that *ref(2)P* polymorphism reflects the progress of a very recent selective sweep, and that in Europe during the 1980s, this was followed by a sweep of a sigma virus strain able to infect flies carrying this mutation. We find that the *ref(2)P* resistance mutation is considerably older than the recent spread of this viral strain and suggest that—possibly because it is recessive—the initial spread of the resistance mutation was very slow.

## 1. Introduction

In natural populations, variation in the ability to resist infection often seems to be mediated by major-effect polymorphisms in single genes. Similarly, pathogen populations contain major-effect polymorphisms that enable them to overcome host resistance. But how are these polymorphisms maintained?

It is possible that such variation is transient and exists because a selective sweep is in progress. After all, it is advantageous for hosts to be resistant and pathogens to be infective, so we might expect alleles that confer resistance or affect pathogen infectivity to spread to fixation. However, advantageous alleles will be fixed rapidly and so we expect these polymorphisms to be rare. Alternatively, such polymorphisms could be maintained by frequency-dependent selection. Haldane (1949, 1954) argued that selection will favour pathogens which are adapted to the most common host genotypes, which, in turn, will confer an advantage to rare host genotypes. Negative frequency-dependent selection can maintain a diversity of both host and pathogen alleles.

Some influential models of frequency-dependent selection have been based on a ‘gene-for-gene concept’, which proposes that for each polymorphic gene that confers pathogenicity in the parasite, there is a corresponding gene that confers a response in the host (Flor 1955). However, despite the central role of gene-for-gene models in evolutionary biology, these interactions have rarely been studied in detail in nature. Most work has been carried out in plant agricultural systems, but the process of selecting for extreme genotypes during breeding and the ecological simplicity of these systems make it difficult to extrapolate these results to natural populations (Sidhu 1984; Barrett 1985; Thompson & Burdon 1992), and it is not known whether results from plants are relevant to other taxa.

We are studying one of the first examples of a gene-for-gene interaction in animals—a simple interaction between *Drosophila melanogaster* and the sigma virus (Brun & Plus 1998). The sigma virus is a common natural pathogen of *D. melanogaster* that reduces the egg viability of infected flies (Fleuriet 1981). Its only mode of transmission is from parent to offspring through sperm and eggs, and it is the only known species-specific pathogen of *D. melanogaster*. Another attractive feature of this model system is that infected flies are paralysed or killed by high concentrations of carbon dioxide (e.g. Coulon & Contamine 1982), providing a simple assay of sigma virus infection in *D. melanogaster*.

Natural populations of *D. melanogaster* contain both susceptible and resistant alleles of a gene called *ref(2)P* (Fleuriet 1988; Contamine *et al*. 1989). *ref(2)P* encodes a protein that sits within the Toll pathway (an important component of the innate immune system) although it is not known what *ref(2)P* does there. Mutations in this gene also affect male fertility (Dezelee *et al*. 1989; Avila *et al*. 2002). Previous studies found that low transmission of the sigma virus is associated with a complex mutation in *ref(2)P* in which CAG–AAT (glutamine–asparagine) has changed to GGA (glycine; Dru *et al*. 1993; Wayne *et al*. 1996). We tested whether this *ref(2)P* mutation was associated with resistance to sigma in *D. melanogaster* collected from a natural population in Pennsylvania, USA. A previous study suggested that different *ref(2)P* resistance alleles confer different degrees of resistance to the sigma virus (Dru *et al*. 1993), although this study did not control fully for the genetic background of the *ref(2)P* gene. We have used a powerful quantitative genetic approach to test whether other polymorphisms in *ref(2)P* affect susceptibility to the sigma virus.

It is thought that the maintenance of the *ref(2)P* resistance/susceptibility polymorphism might reflect an interaction between parasite and host. Two distinct genotypes of the sigma virus have been found in natural populations: ‘infective’ viruses that can infect flies which have the resistant *ref(2)P* allele, and ‘avirulent’ viruses that cannot (Fleuriet 1980). However, it is not known whether these polymorphisms are maintained by frequency-dependent selection or they are transient polymorphisms that exist only while the resistant *ref(2)P* allele and the infective viral strain sweeps to fixation. Several studies favour the idea that the resistance polymorphism is caused by the progress of a transient selective sweep. During the 1980s, there was a dramatic increase in the frequency of the infective viral genotype in some French and German populations, indicating that a selective sweep was occurring (Fleuriet 1980; Fleuriet *et al*. 1990). There is also evidence that selection has promoted amino acid polymorphism within *ref(2)P*—Wayne *et al*. (1996) identified an excess of amino acid polymorphisms among lines (relative to between species) at the 5′ region of the gene (where the complex mutation occurs). This is consistent with both an arms race model in which the resistant allele is currently sweeping to fixation, and frequency-dependent selection maintaining variation in this gene. The sweep model predicts that there will be reduced variation among the resistant alleles (Wayne *et al*. 1996), but only three resistant alleles were sampled in these studies, making it difficult to test this.

We have taken two approaches to investigate how the *ref(2)P* polymorphism is maintained. First, we use a coalescent approach to test whether positive selection has been acting to increase the frequency of the resistance mutation. Second, we have estimated the age of the resistance mutation. If the recent spread of the infective viral genotype has occurred in response to a sweep of the *ref(2)P* resistance mutation, then we might expect that the resistance mutation arose shortly before this. If the polymorphism is much older, then it could have been maintained by frequency-dependent selection. Previously, three resistant *ref(2)P* alleles were found to be similar to susceptible alleles and the resistant alleles were inferred to have arisen recently (Wayne *et al*. 1996). Although this previous study shows that this is not an ancient polymorphism, the data are consistent with allelic ages ranging from a few years to many thousands of years. In the present study, we have used a more powerful method of estimating the age of an allele from linkage disequilibrium with flanking markers.

## 2. Material and methods

### (a) Drosophila melanogaster lines and resistance assays

We used two different sets of fly lines. Eighty-four different second-chromosome substitution lines (created by Lazzaro (2004)) were used for the resistance assay and sequencing the *ref(2)P* gene. Each of these lines contain a different wild-type homozygous second chromosome sampled from a population in Pennsylvania (USA) in 1998 and 1999, which had been substituted into a common isogenic genetic background. To estimate the age of the resistant alleles we needed a larger sample of chromosomes, and for this we also used 169 *D. melanogaster* lines that had been collected by Trudy Mackay from a single population in Raleigh, North Carolina, USA in 2002 and inbred for 20 generations by brother–sister mating. We also tested the frequency of a complex mutation (known to confer resistance to sigma infection) in several additional *D. melanogaster* populations: 24 isofemale lines from each of Gabon, Kenya and Zimbabwe and 23 isofemale lines from The Netherlands.

To measure the effect of *ref(2)P* nucleotide polymorphisms on susceptibility to the sigma virus transmitted from the female parent, we crossed males from the second-chromosome substitution lines to females of the deficiency stock *Df(2L)E55/CyO* (Dru *et al*. 1993). The deficiency stock is infected with the avirulent sigma virus strain A3 (Dru *et al*. 1993). The deficiency is a chromosomal deletion of the region from 37D2-E1 to 37F5-38A1, which includes the *ref(2)P* gene. Therefore, we studied the effects of *ref(2)P* polymorphisms in hemizygotes and heterozygotes.

To rear the *Df(2L)E55/CyO* stock at constant density, we washed eggs off apple juice–agar plates and pipetted 22 μl of eggs to half-pint bottles containing standard maize–sugar–yeast media. The flies were reared at 25°C on a 12 h light/dark cycle and virgin females were collected. Three virgin females that were 3–4 days old were crossed to three males from the chromosome-substitution lines. For each of these lines, an average of 4.25 replicate crosses were set up, giving a total of 370 crosses. These flies were allowed to lay in a vial for 2 days and then tipped into a fresh vial and allowed to lay for another 2 days. The progeny from each vial was collected on two different days to ensure that they were all the same age. Therefore, for each cross there were four sets of offspring. The sigma virus causes infected flies to die or become paralysed on exposure to CO_2_. To estimate the transmission rate of the sigma virus from parent to offspring, the progeny were exposed to pure CO_2_ for 15 min at 13°C (Contamine 1980). After 2 h, the numbers of living and dead progeny were recorded and from this the proportion of infected offspring were calculated. Overall, a total of 45 353 offspring were assayed for infection by the sigma virus.

### (b) Association mapping

We sequenced 2666 bp from all 84 second-chromosome substitution lines. This included 665 bp upstream from the start codon and a 630 bp intron. We are missing the final 711 bp of the 1800 bp of coding sequence described by Wayne (Wayne *et al*. 1996).

We assayed for sigma virus infection in male and female *ref(2)P* hemizygotes and heterozygotes. We calculated the mean proportion of individuals infected with the sigma virus. This provided a measure of resistance to the sigma virus infection of males and females hemizygous and heterozygous for *ref(2)P*, and these four datasets were analysed separately. In calculating the mean proportion, each of the four age replicates was given an equal weight.

The statistical analysis was performed using the R software and language. First, we tested whether resistance was associated with an *a priori* candidate resistance mutation already known to confer the resistance against sigma in other populations. To do this, we performed a one-way analysis of variance using the mean infection rate as a response and the state at a single polymorphic site as a predictor, and from these we calculated the *F*-statistic. The null distribution of the *F*-statistic was generated by permuting the trait values over the genotype and recalculating the *F*-statistic for 10 000 times (Doerge & Churchill 1996). The statistical significance was taken as the proportion of times that the observed *F*-statistic was larger than that calculated from the permuted data.

Second, we tested whether any of the other 47 polymorphic sites were associated with transmission of the sigma virus. An *F*-statistic was calculated for each of the polymorphic sites (again, mean infection rate as a response and state at the site as a predictor). To correct for the effect of multiple tests, an experiment-wide significance threshold of the *F*-statistic was calculated. The mean infection rate was permuted, but this time the *F*-statistic was calculated for each of the polymorphisms in turn, and only the largest of these *F*-values was retained. This was repeated 10 000 times to generate a null distribution of the maximum experiment-wide *F*-statistic. The statistical significance of each polymorphic site was taken as the proportion of times that each observed *F*-statistic was larger than the maximum experiment-wide *F*-statistic.

Finally, we tested whether the *a priori* candidate sigma-resistance mutation was associated with resistance against a range of bacteria. Lazzaro *et al*. (2004, 2006) had measured the susceptibility of the same chromosome-extracted lines to several different bacteria. The permutation analysis was performed as for the *a priori* candidate mutation and sigma-resistance data above.

### (c) Selection on the resistance mutation

If a mutation has a selective advantage by conferring resistance to sigma virus, then that mutation will increase in frequency. This increase in frequency will mean that nucleotide diversity among resistant haplotypes will be low. Hence, to detect whether a recent partial selective sweep of this haplotype has occurred, we tested whether there is less variation among the resistant haplotypes than expected by chance.

We used coalescent simulations (performed using CoaSim (Mailund *et al*. 2005)) to assess the probability that genetic diversity within the resistant haplotype conformed to the expectation under a neutral coalescent.

### (d) The age of the resistance mutation

We estimated the number of generations since the susceptible and resistant alleles in our sample shared a common ancestor by determining the extent of linkage disequilibrium with flanking markers. We identified polymorphisms to use as markers by sequencing several regions near *ref(2)P* on a single resistant and a single susceptible chromosome. Several of these polymorphisms were discarded from our analysis because they were at a low frequency in the population, and this left us with three markers. The first was a 24 bp INDEL 1749 bp downstream from the resistance mutation. This was scored using length differences in a polymerase chain reaction (PCR) product run on an agarose gel. The second was an A/C single-nucleotide polymorphism (SNP) 2823 bp upstream from the mutation. This was scored by digesting a PCR product with the restriction enzyme *Fnu*4H, which only cuts one of the alleles. The third marker was an A/C SNP 9500 bp upstream from the resistance mutation, which was scored by digesting a PCR product with the enzyme *Ssp*I. We scored the resistance mutation itself by digesting a PCR product with the enzyme *Msp*I, which only cuts the resistant allele. The inbred flies from a natural population from North Carolina, USA, were genotyped using these markers and heterozygotes were discarded.

To estimate the age of the mutation from this data, it is necessary to know the rate of recombination between the markers. We used two different estimates of the rate of recombination (crossing events per generation per bp (*c*)), which were obtained by Marais *et al*. (2003) by comparing the genetic and physical maps of chromosome 2 using the methods of Hey & Kliman (2002). These were chosen because they use the most accurate physical map (the genome sequence). The two methods used were the polynomial method (HK-p: *c*=1.04×10^−9^ bp^−1^ per generation in females) and sliding window method (HK-w: *c*=4.62×10^−9^ bp^−1^ per generation in females). To obtain the recombination fraction between two markers, these numbers were divided by two (because there is no recombination in males) and multiplied by the number of nucleotides separating the two markers.

We also obtained an independent estimate of the recombination rate from our DNA sequences using the approximate-likelihood method implemented by the program LDhat (McVean *et al*. 2002). This estimate (LDhat: *c*=1.33×10^−9^ bp^−1^ per generation in females, assuming an effective population size of 10^6^) was very similar to those estimated from comparing the genetic and physical maps. This method is based on a coalescent model which assumes that there has been no selection on the gene. We show below that this assumption is not met in our data. However, selection has only affected the minority of the haplotypes that carry the resistance mutation and, excluding these sequences, it has little effect on this estimate.

## 3. Results

### (a) Polymorphisms associated with resistance

**Figure 1:**
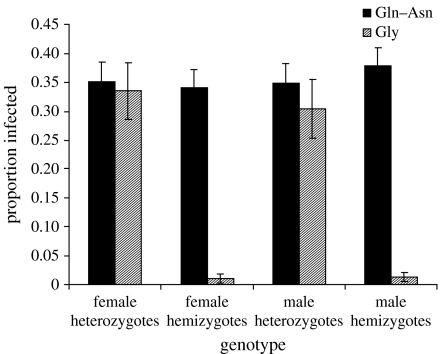
Mean proportion of individuals infected with the sigma virus. Males and females hemizygous for the *ref(2)P* gene (encoding the resistant form, Gly; or the susceptible form, Asn–Gln), and males and females heterozygous for the *ref(2)P* gene (encoding the resistant form, Gly; or the susceptible form, Asn–Gln) are shown.

We identified 41 SNPs and 8 INDEL polymorphisms. One of these SNPs was within an INDEL, so instead of scoring two forms of that SNP, the INDEL had three forms. One of these mutations (a complex mutation in the first exon in which the amino acids Gln and Asn have been replaced by a single Gly) was previously described by Dru *et al*. (1993) and Wayne *et al*. (1996) as conferring resistance to infection by the sigma virus. We found that 19 out of our 84 lines had this allele. In both hemizygous males and females, these 19 lines also had the 19 lowest infection rates (males, *F*=155.1, permutation test *p*<0.0001; females, *F*=118.1, permutation test *p*<0.0001; figure 1). However, there was no association between this polymorphism and infection rates in heterozygotes (males, *F*=1.579, permutation test *p*=0.2097; females, *F*=0.1859, permutation test *p*=0.6678), indicating that the mutation is recessive (figure 1).

We tested for the presence of the resistance mutation from samples all over the world: of 84 samples from Pennsylvania (USA), 19 had this mutation; of 169 samples from North Carolina (USA), 20 had the mutation; of 24 samples from Gabon, none had the mutation; of 24 samples from Kenya, none had the mutation; of 24 samples from Zimbabwe, none had the mutation; and of 23 samples from The Netherlands, none had the mutation. Heterogeneity between populations was analysed in contingency tables. Significance was assessed by comparing the observed data with 200 000 randomly generated contingency tables with the same marginal values, using a Monte Carlo procedure (Lewontin & Felsenstein 1965). This showed that there was significant heterogeneity between populations for the presence of the resistance mutation (*p*<0.0001).

With the exception of a single INDEL, the 19 resistant haplotypes were identical, therefore these haplotypes were removed from the dataset before testing if any of the other polymorphisms in the *ref(2)P* gene were associated with resistance. There was no association between resistance to sigma and any of these other polymorphisms (see the electronic supplementary material).

An allele called *ref(2)P*^*n*^ was reported by Dru *et al*. (1993) to confer resistance to sigma. This allele contains a 21 bp deletion in exon 2 that was absent from all 84 of our sequences. We tested other populations for this deletion by designing primers on either side of it and checking the length of the PCR product on an agarose gel. The deletion was also absent from 96 inbred lines from North Carolina, 24 isofemale lines from Gabon, 24 isofemale lines from Kenya, 24 isofemale lines from Zimbabwe and 23 isofemale lines from The Netherlands.

A previous study (Lazzaro *et al*. 2006) measured the susceptibility of the same second-chromosome extracted lines to several different bacteria. We used their phenotypic data to test whether our sigma-resistance mutation is associated with resistance to bacterial infection. Using the same permutation analysis described above, we found no significant associations between the mutation and resistance to infection by any of the bacteria tested by Lazzaro *et al*. (*Serratia marcescens*, *F*=2.477, permutation test *p*=0.1134; *Providencia burhodogranaria*, *F*=1.831, permutation test *p*=0.1822; *Enterococcus faecalis*, *F*=0.1497, permutation test *p*=0.6967; *Lactococcus lactis*, *F*=3.407, permutation test *p*=0.0640). We carried out similar analysis on a more extensive study that Lazzaro and colleagues had carried out on *S. marcescens* alone (Lazzaro *et al*. 2004), and again we found no significant associations between the resistance mutation and susceptibility to *S. marcescens* infection (data not shown).

### (b) Selection on the resistance mutation

Across the 84 second-chromosome substitution lines, the 2666 bp sequence contained 41 SNPs and 8 INDEL polymorphisms. Across synonymous sites, *π*=0.00119 and *θ*_W_=0.00195 (number of sites: 307.51), and in the coding sequence *π*=0.0023 and *θ*_W_=0.0026. For synonymous sites, the diversity is low when compared with other genes and the mean reported by Andolfatto (2001), which could be due to the low recombination rate in the *ref(2)P* region. The higher diversity at non-synonymous sites could be because selection has inflated diversity within *ref(2)P*—this is consistent with Wayne *et al*. (1996), who observed a significant excess of non-synonymous polymorphisms relative to divergence in the amino-terminal region.

If a mutation has a selective advantage by conferring resistance to sigma virus, then that mutation will increase in frequency. This increase would cause nucleotide diversity among resistant haplotypes to be low. Hence, to find out whether a partial selective sweep of this haplotype had occurred, we used a coalescent approach to test whether there is less variation among the resistant haplotypes than expected by chance. We used coalescent simulations to produce a null distribution of the number of SNPs expected if the resistant alleles conformed to a neutral coalescent. Using an infinite sites model with recombination, we generated coalescent trees for 84 sequences. A trait mutation was added randomly to each tree at the observed position of the resistance mutation (site 743), and trees for which exactly 19 of the 84 haplotypes that carried the resistance mutation were retained. If the trait mutation was at any other frequency, the tree was rejected and a new one generated. Mutations were then added randomly to each tree to give 39 segregating sites, as observed in the data. This rejection sampling procedure was repeated until we had an unbiased sample of 10 000 simulated datasets, each with the resistance mutation at the observed frequency and at the observed position. We then counted the number of segregating sites within the 19 resistant haplotypes in all 10 000 datasets to give a null distribution for the numbers of segregating sites.

In the observed sequences, there were no SNPs among the 19 resistant haplotypes. This was significantly less than expected from our null distribution. Therefore, this haplotype seems to have a selective advantage. The level of significance depends on the recombination rates allowed to happen in the simulation. For the lowest of our three recombination estimates, *p*=0.01, and for the higher recombination estimate, *p*=0.0002. This result is fairly robust to our assumption as it remains significant (*p*<0.05) even if the recombination rate is reduced to one-quarter of our lowest estimate of the recombination rate.

### (c) Age of the resistant allele

When the resistance mutation first arose, it would have been in linkage disequilibrium with flanking markers. We used the extent to which this ancestral chromosome has subsequently recombined with susceptible chromosomes to estimate the age of the mutation. In 169 inbred fly lines from North Carolina, we genotyped the resistance mutation together with two upstream markers (at positions −9500 and −2823 relative to the resistance mutation) and one downstream marker (at position 1749). There was significant linkage disequilibrium between all three of these markers and the resistance mutation (table 1). Therefore, the marker allele that is overrepresented on the resistant chromosomes is presumably the allele that was present on the ancestral chromosome on which the mutation arose. The age of the mutation (*t*) can then be estimated from the frequency of this marker on the resistant chromosomes (*x*), the frequency of this marker on susceptible chromosomes (*y*) and the number of recombination events that have occurred (*r*) between the marker and resistance mutation, using the equation *t*=(1/(ln(1−*r*)))(ln((*x*−*y*)/(1−*y*))) (Serre *et al*. 1990; Slatkin & Rannala 2000).

It is straightforward to estimate *t* using the two markers on either side of *ref(2)P* (at positions −2823 and 1749). We can also obtain a third independent estimate of *t* using the marker at position −9500 by including only recombination events that have occurred between this marker and the marker at position −2823. To do this, we excluded the chromosomes for which there had been a crossing-over event between the resistance mutation and the −2823 mutation. We can detect these recombinants with reasonable certainty because the allele at −2823 that is associated with the resistance mutation is totally absent from our sample of 137 susceptible chromosomes (table 1). In the remaining chromosomes, our estimate of *x* based on the −9500 marker is now only affected by recombination between the markers at −2823 and −9500. By also calculating *r* between these two markers, we can now obtain a third independent estimate of *t*.

The estimated age of the mutation (*t*) ranges from 22 000 to 152 000 generations (which, with approximately 20 generations per year, equates to approximately 1000–7000 years). The main source of variation between these estimates arises from the different methods used to calculate the recombination rate. A second source of error in our estimates arises from the stochastic nature of recombination, but it is not possible to calculate accurate confidence limits for *t* because our results indicate that *ref(2)P* has been under strong selection pressure. We do not know what these selection pressures have been, so we are unable to model them and obtain meaningful confidence limits for *t*. However, we have provided three independent estimates of *t* and these are reasonably consistent. Furthermore, it is worth noting that it is to our advantage that *D. melanogaster* does not appear to have recombination hotspots (Andolfatto & Przeworski 2000). Therefore, it seems clear that the stochasticity of recombination is a less important source of uncertainty than that arising from the different estimates of the recombination rate (table 1). It should be noted that we are estimating the time since the alleles in our sample shared a common ancestor. The mutation itself may be older than this, especially if it first occurred on a different continent and only recently spread to North America.

## 4. Discussion

We found that a mutation within *ref(2)P* that was already known to confer resistance to the sigma virus was present in 19 of our 84 samples of wild-type second chromosomes. This allele contains a major-effect complex mutation, whereby CAG–AAT has changed to GGA, replacing Gln–Asn with a single Gly, which must have involved more than one event (a minimum of one insertion and one deletion; Wayne *et al*. 1996). We confirmed that this mutation is correlated with resistance to sigma virus infection transmitted from a female parent to offspring; indeed, this mutation explains much of the genetic variation in resistance in this population. This confirms the results of a smaller study in which sequences of *ref(2)P* from 13 strains of *D. melanogaster* identified this mutation as responsible for resistance to sigma in three of the strains (Wayne *et al*. 1996). Our data strengthens this previous result, which was not a single controlled experiment, but was based on trait measurements made in different laboratories at different times (Wayne *et al*. 1996).

Do other polymorphisms affect resistance to sigma? It has been suggested that there are additional polymorphisms in *ref(2)P* that increase the ‘strength’ of resistant alleles (Dru *et al*. 1993), so we examined the effects of the remaining 47 polymorphisms in the *ref(2)P* coding sequence and upstream region in our chromosome-extracted lines. None of the other polymorphisms affected the transmission of the sigma virus.

Why were we were unable to find evidence for the different resistance alleles described previously (Dru *et al*. 1993)? Although we were unable to sequence the final 711 bp of the coding region of *ref(2)P*, the additional ‘resistance’ allele reported by Dru *et al*. (1993) contained no unique polymorphisms in this region of the gene. One possibility is that the differences seen in the previous study were caused by other nearby genes. Dru *et al*. (1993) introgressed two different *ref(2)P* alleles into a common genetic background and measured the resistance of these flies to the sigma virus—this protocol will have left an average of approximately 17 Mb of surrounding chromosome linked to *ref(2)P*, and polymorphisms anywhere in this region could alter resistance. By contrast, our association approach used natural populations that have undergone thousands of generations of recombination, which makes the uncontrolled region around the gene much smaller (as is illustrated by the decline in linkage disequilibrium that we saw over a few kilobases, table 1). Alternatively, the additional *ref(2)P* allele described by Dru *et al*. could be missing from our dataset. Indeed, the allele had a 21 bp deletion that we found to be absent from our chromosome-extracted lines. It is also missing from samples of another USA population, a European population and three African populations, so this allele is unlikely to be an important cause of natural genetic variation.

Can we say anything about the models of coevolution that best describe the selection acting on this gene? Models of host–parasite coevolution fall into two main classes. Arms-race models propose that new host resistance or parasite virulence mutations arise and sweep to fixation under directional selection. Under this scenario, resistance polymorphisms are transient, existing only during the sweep. Frequency-dependent models state that selection favours pathogens adapted to the most common host genotypes, and that this, in turn, confers an advantage to rare host genotypes.

We investigated whether positive selection has acted on the *ref(2)P* resistance mutation by testing whether there was reduced variation among the resistant haplotypes. Because the *ref(2)P* resistance mutation is at low frequency in the population (23%), standard haplotype tests of neutral evolution (Innan *et al*. 2005) were not powerful enough to detect a reduction in diversity (data not shown). The power of our approach came from knowing which mutation (the resistance mutation) was under selection—so that under the neutral scenario, each genealogy created contained 19 sequences with a mutation in the observed position of the resistance mutation. By comparing the observed number of SNPs among the resistant haplotypes with the number of segregating sites in the resistant haplotypes under the neutral scenario, we showed that there was less variation among the resistant haplotypes than expected by chance. Our results indicate that positive selection has increased the frequency of the resistance mutation. Our finding extends the results of a previous study that found an excess of replacement polymorphisms at the 5′ end of the gene (Wayne *et al*. 1996), but which only used three resistant haplotypes, which was too few to test for reduced variation.

Positive selection having acted on the gene is consistent with a selective sweep. But could such a sweep have occurred during the last 30 years? If an arms race between *ref(2)P* and sigma had caused a recent sweep, then we might expect the spread of the ‘infective’ viral genotype to have occurred immediately after the spread of the resistant *ref(2)P* mutation. To test this, we determined the age of this mutation by calculating the degree of linkage with markers flanking the *ref(2)P* gene. This showed the resistance mutation to be several thousand years old. There are several uncertainties associated with estimating the precise age of the allele (Slatkin & Rannala 2000) but it is clear that although the resistance mutation is not ancient, it long predates the spread of the infective virus.

How can we reconcile the finding that the resistant *ref(2)P* mutation emerged several thousand years ago, with the recent spread of a viral type that can infect flies carrying this mutation? First, it is worth remembering that our population of flies came from the USA, whereas the studies on the viral sweep were in Germany and France, and populations on the two continents might have been affected by different selective pressures. However, there are other possible explanations. It is possible that an arms race is going on between *ref(2)P* but that the *ref(2)P* resistance gene has only recently become frequent enough to select for viral countermeasures. Figure 1 indicates that our mutation is mostly recessive, which means it could take thousands of generations to reach the current frequency. In samples collected from populations across three different continents, the frequency of the resistance mutation has never exceeded 23%, so only 5% of flies will be homozygous and therefore resistant to infection.

Second, it is possible that the mutation arose several thousand years ago and has since been maintained by frequency-dependent selection. If this system represented a gene-for-gene scenario (Agrawal & Lively 2002), we would expect that this mutation carries a cost. What costs might the resistance mutation carry? One possibility is that the resistance mutation in *ref(2)P* carries with it a fertility cost. Knocking out *ref(2)P* completely causes male sterility (Dezelee *et al*. 1989), so it is possible that the protein is involved in gamete formation. Another possibility is that it compromises the fly's ability to fight other infections. Ref(2)P is also involved in the Toll pathway (Avila *et al*. 2002), which is a crucial part of the fly's immune defence and the ‘resistance’ mutation could compromise defence against other pathogens. We have tested this last hypothesis using the phenotypic infection data generated by Lazzaro *et al*. (2004, 2006) for the same lines as used in the present study. Lazzaro and colleagues tested the susceptibility of the 2nd chromosome-extracted lines to two Gram-positive bacteria and two Gram-negative bacteria. We found no association between the presence of the *ref(2)P* resistance mutation and susceptibility to infection by these bacteria, so there is no evidence that this is a cause of a cost associated with this mutation.

In conclusion, a change of just two amino acids in the *ref(2)P* protein makes flies resistant to the sigma virus. Our results indicate that this mutation arose several thousand years ago and spread because it had a selective advantage. The data show that the resistant allele is largely recessive, so we suggest that its initial spread will have been slow and it may only have become common very recently. Within the last 20 years, a viral strain that can infect resistant flies has swept across Europe (Fleuriet 1980; Fleuriet *et al*. 1990). While we cannot reject the hypothesis that negative frequency-dependent selection maintains the resistance polymorphism, the simplest explanation of our results is that this is a transient polymorphism.

## Figures and Tables

**Table 1:** The age of the resistance mutation estimated from marker frequencies on the resistant and susceptible chromosomes.

marker location relative to mutation (bp)	resistant chromosome		susceptible chromosome		age of mutation (generations)			
		
	number of chromosomes with marker type A	number of chromosomes with marker type B	number of chromosomes with marker type A	number of chromosomes with marker type B	*p*^a^	polynomial^b^	sliding window^b^	LDdhat^b^
1749	19	1	56	73	<0.0001	101 518	22 852	79 535
−2823	12	3	0	137	<0.0001	151 717	34 153	118 864
−9500	14	5	52	77	0.0059			
−9500^c^	13	4	52	77	0.0043	144 075	32 432	112 876

a The significance of the difference in marker frequency on susceptible and resistant chromosomes calculated using a *G*-test.

b Three different methods used to estimate the recombination rate.

c Excludes chromosomes that have recombined between *ref(2)P* and marker −2832 (see text). Note that some chromosomes were heterozygous, which means that the number of resistant chromosomes does not always add up to 20 and the number of susceptible chromosomes does not always add up to 129.

## Supplementary Material

Table of polymorphisms for each chromosome-extracted line Mean proportion of infected individuals of each chromosome-extracted line, for male and female heterozygotes and hemizygotes. Also shows genotypes and positions of the 41 SNPs and 8 INDEL polymorphisms. One of these SNPs was within an INDEL, so instead of scoring two forms of that SNP, the INDEL had three forms
