# Supplementary material for: The age and evolution of an antiviral resistance mutation in Drosophila melanogaster
Source: Proc Biol Sci. 2007 Jun 5;274(1621):2027–34. doi: 10.1098/rspb.2007.0611 (PMC1914336; doi:10.1098/rspb.2007.0611)
Supplement: Supplementary file 1 [file rspb20070611s11.pdf]

	Hemizygotes		Heterozygotes		Polymorphisms (position relative to start codon)					Exon 1					Intron 1																								Exon 2														
Line	Female	Male	Female	Male	-556	-494	-388	-169	-80	82	94	95	128	236	288	301	357	369	424	431	466	490	494	500	535	536	541	545	568	572	599	661	672	706	734	801	824	841	864	898	984	1354	1403	1484	1532	1654	1730	1737	1828	1841	1861	1994
10H	0.318	0.375	0.410	0.330	A	DEL	T	A	A	CAGAAT	A	T	A	INS	T	C	C	DEL	T	INS	C	GG	A	AA	C	A	T	A	G	INS	C	A	INS	G	C	C	A	G	C	T	C	T	C	C	T	C	C	A	C	T	INS1	G
13C	0.481	0.406	0.469	0.545	.	.	.	.	.	.	.	.	.	.	.	.	.	.	.	.	.	.	.	.	.	.	.	.	.	.	.	.	.	.	.	.	.	.	.	.	.	.	.	.	.	.	.	.	.	.	.	.
1H	0.159	0.377	0.289	0.286	.	.	.	.	.	.	.	.	.	.	.	.	.	.	.	.	.	.	.	.	.	.	.	.	.	.	.	.	.	.	.	.	.	.	.	.	.	.	.	.	.	.	.	.	.	.	.	.
3B	0.348	0.406	0.320	0.481	.	.	.	.	C	.	G	C	T	.	A	.	T	INS	.	.	T	DEL	T	GG	.	C	A	C	T	DEL	A	G	DEL	.	.	A	T	.	A	.	.	.	.	.	.	.	.	.	.	C	.	T
3H	0.473	0.519	0.619	0.593	.	.	.	.	.	.	.	.	.	.	.	.	.	.	.	.	.	.	.	.	.	.	.	.	.	.	.	.	.	.	.	.	.	.	.	.	.	.	.	.	.	.	.	.	.	.	.	.
4B	0.274	0.340	0.436	0.360	.	.	.	.	.	.	.	.	.	.	.	.	.	.	.	.	.	.	.	.	.	.	.	.	.	.	.	.	.	.	.	.	.	.	.	.	.	.	.	.	.	.	.	.	.	.	.	.
5E	0.323	0.330	0.380	0.269	.	.	.	.	.	.	.	.	.	.	.	.	.	.	.	.	.	.	.	.	.	.	.	.	.	.	.	.	.	.	.	.	.	.	.	.	.	.	.	.	.	.	.	.	.	.	.	.
7B	0.111	0.303	0.243	0.258	.	.	.	.	.	.	.	.	.	.	.	.	.	.	.	.	.	.	.	.	.	.	.	.	.	.	.	.	.	.	.	.	.	.	.	.	.	.	.	.	.	.	.	.	.	.	.	.
7H	0.507	0.564	0.508	0.501	.	.	.	.	.	.	.	.	.	.	.	.	.	.	.	.	.	.	.	.	.	.	.	.	.	.	.	.	.	.	.	.	.	.	.	.	.	.	.	.	.	.	.	.	.	.	.	.
8F	0.435	0.424	0.288	0.411	.	.	.	.	C	.	G	C	T	.	A	.	T	INS	.	.	T	DEL	T	GG	.	C	A	C	T	DEL	A	G	DEL	.	.	A	T	.	A	.	.	.	.	.	.	.	.	.	.	C	.	T
8G	0.448	0.580	0.512	0.604	.	.	.	.	.	.	.	.	.	.	.	.	.	.	.	.	.	.	.	.	.	.	.	.	.	.	.	.	.	.	.	.	.	.	.	.	.	.	.	.	.	.	.	.	.	.	.	.
10A	0.112	0.109	0.099	0.129	G	.	C	.	C	.	.	.	.	DEL	A	.	T	INS	.	.	T	DEL	T	GG	T	C	A	C	T	DEL	A	G	DEL	.	.	A	T	.	.	.	.	A	A	.	.	.	.	T	.	C	.	.
10C	0.369	0.520	0.328	0.436	G	.	C	.	C	.	G	C	T	.	.	.	T	INS	.	.	T	DEL	T	GG	.	C	A	C	T	DEL	A	G	DEL	A	.	A	T	.	.	.	.	.	.	.	.	.	.	.	.	C	DEL	.
10E	0.411	0.328	0.298	0.238	G	.	C	.	C	.	G	C	T	.	.	.	T	INS	.	.	T	DEL	T	GG	.	C	A	C	T	DEL	A	G	DEL	A	.	A	T	T	.	.	.	.	.	.	.	.	.	.	.	C	DEL	.
11B	0.169	0.256	0.142	0.152	G	.	C	.	C	.	G	C	T	.	.	.	T	INS	.	.	T	DEL	T	GG	.	C	A	C	T	DEL	A	G	DEL	A	.	A	T	.	.	.	.	.	.	.	.	.	.	.	.	C	DEL	.
11C	0.304	0.278	0.312	0.442	G	.	C	.	C	.	G	C	T	.	.	.	T	INS	.	.	T	DEL	T	GG	.	C	A	C	T	DEL	A	G	DEL	A	.	A	T	T	.	.	.	.	.	.	.	.	.	.	.	C	INS2	.
11E	0.370	0.358	0.299	0.279	G	INS	C	.	C	.	G	C	T	.	.	.	T	INS	.	.	T	DEL	T	GG	T	C	A	C	T	DEL	A	G	DEL	.	.	A	T	.	.	.	.	A	A	.	.	.	.	T	.	C	.	.
11F	0.574	0.574	0.559	0.455	G	.	C	.	C	.	G	C	T	.	.	.	T	INS	.	.	T	DEL	T	GG	.	C	A	C	T	DEL	A	G	DEL	A	.	A	T	T	.	.	.	.	.	.	.	.	T	.	.	C	DEL	.
11G	0.357	0.345	0.344	0.378	G	.	C	.	C	.	G	C	T	.	.	.	T	INS	.	.	T	DEL	T	GG	.	C	A	C	T	DEL	A	G	DEL	A	.	A	T	.	.	.	.	.	.	.	.	.	.	.	.	C	DEL	.
11H	0.392	0.521	0.357	0.433	G	.	C	.	C	.	G	C	T	.	.	.	T	INS	.	.	T	DEL	T	GG	.	C	A	C	T	DEL	A	G	DEL	A	.	A	T	T	.	.	.	.	.	.	.	.	.	.	.	C	DEL	.
12A	0.243	0.279	0.257	0.251	G	.	C	.	C	.	G	C	T	.	.	.	T	INS	.	.	T	DEL	T	GG	.	C	A	C	T	DEL	A	G	DEL	A	.	A	T	T	.	.	.	.	.	.	.	.	T	.	.	C	DEL	.
12E	0.390	0.610	0.611	0.611	G	.	C	.	C	.	.	.	.	DEL	A	.	T	INS	.	.	T	DEL	T	GG	T	C	A	C	T	DEL	A	G	DEL	.	.	A	T	.	.	.	.	A	.	.	.	A	.	T	.	C	.	.
12F	0.100	0.089	0.074	0.137	G	.	C	.	C	.	.	.	.	DEL	A	.	T	INS	.	.	T	DEL	T	GG	T	C	A	C	T	DEL	A	G	DEL	.	.	A	T	.	.	.	.	A	.	.	.	.	.	T	.	C	.	.
13D	0.271	0.348	0.280	0.274	G	.	C	.	C	.	G	C	T	.	.	.	T	INS	.	DEL	T	DEL	T	GG	.	C	A	C	T	DEL	A	G	DEL	A	.	A	T	.	.	C	T	.	.	.	.	.	.	.	.	C	DEL	.
1A	0.515	0.378	0.525	0.346	G	.	C	.	C	.	G	C	T	.	.	.	T	INS	.	.	T	DEL	T	GG	.	C	A	C	T	DEL	A	G	DEL	A	.	A	T	T	.	.	.	.	.	.	.	.	.	.	.	C	DEL	.
1B	0.164	0.278	0.173	0.193	G	.	C	.	C	.	G	C	T	.	.	.	T	INS	.	DEL	T	DEL	T	GG	.	C	A	C	T	DEL	A	G	DEL	A	A	A	T	.	.	C	T	.	.	.	.	.	.	.	.	C	DEL	.
1C	0.551	0.636	0.734	0.591	G	.	C	G	C	.	.	.	.	.	.	.	.	.	.	.	.	.	.	.	.	.	.	.	.	.	.	.	.	.	.	.	.	.	.	.	.	.	.	.	.	.	.	.	G	C	.	.
1E	0.441	0.424	0.407	0.426	G	.	C	.	C	.	.	.	.	.	.	.	.	.	.	.	.	.	.	.	.	C	A	C	T	DEL	A	G	DEL	.	.	A	T	.	.	.	.	.	.	T	.	.	.	.	.	C	.	.
1F	0.159	0.125	0.217	0.157	G	.	C	.	C	.	.	.	.	DEL	A	.	T	INS	.	.	T	DEL	T	GG	T	C	A	C	T	DEL	A	G	DEL	.	.	A	T	.	.	.	.	A	.	.	.	.	.	T	.	C	.	.
1G	0.167	0.156	0.316	0.134	G	.	C	.	C	.	G	C	T	.	.	.	T	INS	A	.	T	DEL	T	GG	.	C	A	C	T	DEL	A	G	DEL	A	.	A	T	T	.	.	.	.	.	.	.	.	.	.	.	C	DEL	.
2A	0.378	0.373	0.351	0.339	G	.	C	.	C	.	G	C	T	.	.	.	T	INS	.	.	T	DEL	T	GG	.	C	A	C	T	DEL	A	G	DEL	A	.	A	T	T	.	.	.	.	.	.	.	.	.	.	.	C	DEL	.
2C	0.235	0.335	0.196	0.343	G	.	C	.	C	.	G	C	T	.	.	.	T	INS	.	.	T	DEL	T	GG	.	C	A	C	T	DEL	A	G	DEL	A	.	A	T	.	.	.	.	.	.	.	.	.	.	.	.	C	DEL	.
2D	0.490	0.421	0.498	0.513	G	.	C	.	C	.	G	C	T	.	.	.	T	INS	.	.	T	DEL	T	GG	.	C	A	C	T	DEL	A	G	DEL	A	.	A	T	.	.	.	.	.	.	.	.	.	.	.	.	C	DEL	.
2E	0.303	0.394	0.296	0.343	G	.	C	.	C	.	G	C	T	.	.	.	T	INS	.	.	T	DEL	T	GG	.	C	A	C	T	DEL	A	G	DEL	A	.	A	T	.	.	.	.	.	.	.	.	.	.	.	.	C	DEL	.
2F	0.481	0.532	0.411	0.381	G	.	C	.	C	.	.	.	.	DEL	A	.	T	INS	.	.	T	DEL	T	GG	T	C	A	C	T	DEL	A	G	DEL	.	.	A	T	.	.	.	.	A	.	.	.	.	.	T	.	C	.	.
2G	0.230	0.311	0.210	0.155	G	.	C	.	C	.	G	C	T	.	.	.	T	INS	.	.	T	DEL	T	GG	.	C	A	C	T	DEL	A	G	DEL	A	.	A	T	T	.	.	.	.	.	.	.	.	.	.	.	C	DEL	.
2H	0.196	0.221	0.252	0.193	G	.	C	.	C	.	G	C	T	.	.	.	T	INS	.	.	T	DEL	T	GG	.	C	A	C	T	DEL	A	G	DEL	A	.	A	T	T	.	.	.	.	.	.	.	.	.	.	.	C	DEL	.
3C	0.380	0.448	0.316	0.309	G	.	C	.	C	.	G	C	T	.	.	.	T	INS	A	.	T	DEL	T	GG	.	C	A	C	T	DEL	A	G	DEL	A	.	A	T	T	.	.	.	.	.	.	.	.	.	.	.	C	DEL	.
3D	0.463	0.418	0.469	0.508	G	.	C	.	C	.	G	C	T	.	.	.	T	INS	.	.	T	DEL	T	GG	.	C	A	C	T	DEL	A	G	DEL	A	.	A	T	.	.	.	.	.	.	.	.	.	.	.	.	C	DEL	.
3G	0.428	0.453	0.284	0.356	G	.	C	.	C	.	.	.	.	DEL	A	.	T	INS	.	.	T	DEL	T	GG	.	C	A	C	T	DEL	A	G	DEL	.	.	A	T	.	.	.	.	.	.	.	A	.	.	.	.	C	.	.
4A	0.372	0.321	0.281	0.327	G	.	C	.	C	.	G	C	T	.	.	.	T	INS	.	.	T	DEL	T	GG	.	C	A	C	T	DEL	A	G	DEL	A	.	A	T	T	.	.	.	.	.	.	.	.	.	.	.	C	DEL	.
4C	0.227	0.334	0.212	0.220	G	.	C	.	C	.	G	C	T	.	.	.	T	INS	A	.	T	DEL	T	GG	.	C	A	C	T	DEL	A	G	DEL	A	.	A	T	T	.	.	.	.	.	.	.	.	.	.	.	C	DEL	.
4D	0.403	0.321	0.437	0.226	G	.	C	.	C	.	.	.	.	DEL	A	.	T	INS	.	.	T	DEL	T	GG	T	C	A	C	T	DEL	A	G	DEL	.	.	A	T	.	.	.	.	A	.	.	.	A	.	T	.	C	.	.
4E	0.205	0.225	0.132	0.238	G	.	C	.	C	.	G	C	T	.	.	.	T	INS	.	.	T	DEL	T	GG	.	C	A	C	T	DEL	A	G	DEL	A	.	A	T	.	.	.	.	.	.	.	.	.	.	.	.	C	DEL	.
4G	0.437	0.348	0.310	0.369	G	.	C	.	C	.	G	C	T	.	.	.	T	INS	.	.	T	DEL	T	GG	.	C	A	C	T	DEL	A	G	DEL	A	.	A	T	T	.	.	.	.	.	.	.	.	.	.	.	C	DEL	.
4H	0.473	0.685	0.624	0.649	G	.	C	.	C	.	.	.	.	DEL	A	.	T	INS	.	.	T	DEL	T	GG	T	C	A	C	T	DEL	A	G	DEL	.	.	A	T	.	.	.	.	A	.	.	.	A	.	T	.	C	.	.
5B	0.555	0.542	0.576	0.465	G	.	C	.	C	.	.	.	.	DEL	A	.	T	INS	C	.	T	DEL	T	GG	T	C	A	C	T	DEL	A	G	DEL	.	.	A	T	.	.	.	.	A	.	.	.	.	.	.	.	C	DEL	.
5G	0.405	0.516	0.368	0.491	G	.	C	.	C	.	G	C	T	.	.	.	T	INS	.	.	T	DEL	T	GG	.	C	A	C	T	DEL	A	G	DEL	A	.	A	T	.	.	.	.	.	.	.	.	.	.	.	.	C	DEL	.
5H	0.125	0.334	0.334	0.156	G	.	C	.	C	.	G	C	T	.	.	.	T	INS	A	.	T	DEL	T	GG	.	C	A	C	T	DEL	A	G	DEL	A	.	A	T	T	.	.	.	.	.	.	.	.	.	.	.	C	DEL	.
6A	0.483	0.471	0.595	0.486	G	.	C	.	C	.	G	C	T	.	.	.	T	INS	.	.	T	DEL	T	GG	.	C	A	C	T	DEL	A	G	DEL	A	.	A	T	.	.	.	.	.	.	.	.	.	.	.	.	C	DEL	.
6B	0.405	0.423	0.345	0.386	G	.	C	.	C	.	G	C	T	.	.	.	T	INS	.	.	T	DEL	T	GG	.	C	A	C	T	DEL	A	G	DEL	A	.	A	T	.	.	.	.	.	.	.	.	.	.	.	.	C	DEL	.
6F	0.410	0.320	0.364	0.266	G	.	C	.	C	.	G	C	T	.	.	.	T	INS	.	.	T	DEL	T	GG	.	C	A	C	T	DEL	A	G	DEL	A	.	A	T	.	.	.	.	.	.	.	.	.	.	.	.	C	DEL	.
6H	0.447	0.365	0.362	0.407	G	.	C	.	C	.	G	C	T	.	.	.	T	INS	.	.	T	DEL	T	GG	.	C	A	C	T	DEL	A	G	DEL	A	.	A	T	T	.	.	.	.	.	.	.	.	T	.	.	C	DEL	.
7A	0.362	0.316	0.325	0.410	G	.	C	.	C	.	.	.	.	DEL	A	.	T	INS	.	.	T	DEL	T	GG	T	C	A	C	T	DEL	A	G	DEL	.	.	A	T	.	.	.	.	A	A	.	.	.	.	T	.	C	.	.
7C	0.360	0.461	0.262	0.290	G	.	C	.	C	.	G	C	T	.	.	T	T	INS	.	.	T	DEL	T	GG	.	C	A	C	T	DEL	A	G	DEL	A	.	A	T	T	.	.	.	.	.	.	.	.	T	.	.	C	DEL	.
7E	0.279	0.414	0.326	0.288	G	.	C	.	C	.	G	C	T	.	.	.	T	INS	.	.	T	DEL	T	GG	.	C	A	C	T	DEL	A	G	DEL	A	.	A	T	.	.	.	.	.	.	.	.	.	.	.	.	C	DEL	.
7G	0.108	0.223	0.162	0.147	G	.	C	.	C	.	.	.	.	DEL	A	.	T	INS	C	.	T	DEL	T	GG	T	C	A	C	T	DEL	A	G	DEL	.	.	A	T	.	.	.	.	A	.	.	.	.	.	.	.	C	DEL	.
8B	0.256	0.271	0.285	0.197	G	.	C	.	C	.	.	.	.	DEL	A	.	T	INS	.	.	T	DEL	T	GG	T	C	A	C	T	DEL	A	G	DEL	.	.	A	T	.	.	.	.	A	.	.	.	.	.	T	.	C	.	.
8D	0.513	0.499	0.454	0.470	G	.	C	.	C	.	G	C	T	.	.	.	T	INS	.	.	T	DEL	T	GG	.	C	A	C	T	DEL	A	G	DEL	A	.	A	T	.	.	.	.	.	.	.	.	.	.	.	.	C	DEL	.
8E	0.309	0.411	0.179	0.273	G	.	C	.	C	.	G	C	T	.	.	.	T	INS	.	.	T	DEL	T	GG	.	C	A	C	T	DEL	A	G	DEL	A	.	A	T	.	.	.	.	.	.	.	.	.	.	.	.	C	DEL	.
8H	0.513	0.381	0.650	0.572	G	.	C	.	C	.	.	.	.	DEL	A	.	T	INS	.	.	T	DEL	T	GG	T	C	A	C	T	DEL	A	G	DEL	.	.	A	T	.	.	.	.	A	.	.	.	.	.	T	.	C	.	.
9A	0.281	0.367	0.258	0.338	G	.	C	.	C	.	G	C	T	.	.	.	T	INS	.	.	T	DEL	T	GG	.	C	A	C	T	DEL	A	G	DEL	A	.	A	T	.	.	.	.	.	.	.	.	.	.	.	.	C	DEL	.
9C	0.388	0.500	0.388	0.443	G	.	C	.	C	.	G	C	T	.	.	.	T	INS	.	.	T	DEL	T	GG	.	C	A	C	T	DEL	A	G	DEL	A	.	A	T	.	.	.	.	.	.	.	.	.	.	.	.	C	DEL	.
9D	0.219	0.282	0.341	0.308	G	.	C	.	C	.	.	.	.	.	.	.	T	INS	.	.	T	DEL	T	GG	.	C	A	C	T	DEL	A	G	DEL	A	.	A	T	.	.	.	.	A	A	.	.	.	.	T	.	C	.	.
9F	0.072	0.138	0.124	0.089	G	.	C	.	C	.	G	C	T	.	.	.	T	INS	.	DEL	T	DEL	T	GG	.	C	A	C	T	DEL	A	G	DEL	A	A	A	T	.	.	C	T	.	.	.	.	.	.	.	.	C	DEL	.
10D	0.000	0.026	0.245	0.234	G	.	C	.	C	GGA	G	.	T	.	.	.	T	INS	.	.	T	DEL	T	GG	.	C	A	C	T	DEL	A	G	DEL	A	.	A	T	.	.	.	.	.	.	.	.	.	.	.	.	C	DEL	.
10G	0.000	0.000	0.263	0.228	G	.	C	.	C	GGA	G	.	T	.	.	.	T	INS	.	.	T	DEL	T	GG	.	C	A	C	T	DEL	A	G	DEL	A	.	A	T	.	.	.	.	.	.	.	.	.	.	.	.	C	DEL	.
11D	0.006	0.006	0.311	0.281	G	.	C	.	C	GGA	G	.	T	.	.	.	T	INS	.	.	T	DEL	T	GG	.	C	A	C	T	DEL	A	G	DEL	A	.	A	T	.	.	.	.	.	.	.	.	.	.	.	.	C	DEL	.
12B	0.000	0.000	0.214	0.211	G	.	C	.	C	GGA	G	.	T	.	.	.	T	INS	.	.	T	DEL	T	GG	.	C	A	C	T	DEL	A	G	DEL	A	.	A	T	.	.	.	.	.	.	.	.	.	.	.	.	C	.	.
13A	0.044	0.000	0.308	0.280	G	.	C	.	C	GGA	G	.	T	.	.	.	T	INS	.	.	T	DEL	T	GG	.	C	A	C	T	DEL	A	G	DEL	A	.	A	T	.	.	.	.	.	.	.	.	.	.	.	.	C	DEL	.
13F	0.007	0.012	0.342	0.303	G	.	C	.	C	GGA	G	.	T	.	.	.	T	INS	.	.	T	DEL	T	GG	.	C	A	C	T	DEL	A	G	DEL	A	.	A	T	.	.	.	.	.	.	.	.	.	.	.	.	C	DEL	.
1D	0.000	0.009	0.492	0.500	G	.	C	.	C	GGA	G	.	T	.	.	.	T	INS	.	.	T	DEL	T	GG	.	C	A	C	T	DEL	A	G	DEL	A	.	A	T	.	.	.	.	.	.	.	.	.	.	.	.	C	.	.
2B	0.012	0.029	0.147	0.225	G	.	C	.	C	GGA	G	.	T	.	.	.	T	INS	.	.	T	DEL	T	GG	.	C	A	C	T	DEL	A	G	DEL	A	.	A	T	.	.	.	.	.	.	.	.	.	.	.	.	C	DEL	.
3E	0.000	0.008	0.323	0.272	G	.	C	.	C	GGA	G	.	T	.	.	.	T	INS	.	.	T	DEL	T	GG	.	C	A	C	T	DEL	A	G	DEL	A	.	A	T	.	.	.	.	.	.	.	.	.	.	.	.	C	DEL	.
3F	0.016	0.000	0.473	0.458	G	.	C	.	C	GGA	G	.	T	.	.	.	T	INS	.	.	T	DEL	T	GG	.	C	A	C	T	DEL	A	G	DEL	A	.	A	T	.	.	.	.	.	.	.	.	.	.	.	.	C	DEL	.
5A	0.000	0.048	0.241	0.217	G	.	C	.	C	GGA	G	.	T	.	.	.	T	INS	.	.	T	DEL	T	GG	.	C	A	C	T	DEL	A	G	DEL	A	.	A	T	.	.	.	.	.	.	.	.	.	.	.	.	C	DEL	.
5D	0.000	0.042	0.490	0.474	G	.	C	.	C	GGA	G	.	T	.	.	.	T	INS	.	.	T	DEL	T	GG	.	C	A	C	T	DEL	A	G	DEL	A	.	A	T	.	.	.	.	.	.	.	.	.	.	.	.	C	DEL	.
5F	0.000	0.012	0.348	0.296	G	.	C	.	C	GGA	G	.	T	.	.	.	T	INS	.	.	T	DEL	T	GG	.	C	A	C	T	DEL	A	G	DEL	A	.	A	T	.	.	.	.	.	.	.	.	.	.	.	.	C	DEL	.
6D	0.013	0.000	0.343	0.317	G	.	C	.	C	GGA	G	.	T	.	.	.	T	INS	.	.	T	DEL	T	GG	.	C	A	C	T	DEL	A	G	DEL	A	.	A	T	.	.	.	.	.	.	.	.	.	.	.	.	C	DEL	.
6E	0.013	0.009	0.303	0.257	G	.	C	.	C	GGA	G	.	T	.	.	.	T	INS	.	.	T	DEL	T	GG	.	C	A	C	T	DEL	A	G	DEL	A	.	A	T	.	.	.	.	.	.	.	.	.	.	.	.	C	.	.
7F	0.000	0.000	0.317	0.205	G	.	C	.	C	GGA	G	.	T	.	.	.	T	INS	.	.	T	DEL	T	GG	.	C	A	C	T	DEL	A	G	DEL	A	.	A	T	.	.	.	.	.	.	.	.	.	.	.	.	C	DEL	.
8A	0.063	0.000	0.538	0.542	G	.	C	.	C	GGA	G	.	T	.	.	.	T	INS	.	.	T	DEL	T	GG	.	C	A	C	T	DEL	A	G	DEL	A	.	A	T	.	.	.	.	.	.	.	.	.	.	.	.	C	DEL	.
8C	0.014	0.000	0.213	0.127	G	.	C	.	C	GGA	G	.	T	.	.	.	T	INS	.	.	T	DEL	T	GG	.	C	A	C	T	DEL	A	G	DEL	A	.	A	T	.	.	.	.	.	.	.	.	.	.	.	.	C	DEL	.
9H	0.000	0.054	0.454	0.362	G	.	C	.	C	GGA	G	.	T	.	.	.	T	INS	.	.	T	DEL	T	GG	.	C	A	C	T	DEL	A	G	DEL	A	.	A	T	.	.	.	.	.	.	.	.	.	.	.	.	C	DEL	.
